# Johnson County Medical Society

**Published:** 1890-03

**Authors:** T. C. Osborn

**Affiliations:** Sec’y J. C. M. S.


### Society Notes.

### JOHNSON COUNTY MEDICAL SOCIETY.

At a recent meeting of this flourishing Society, the members agreed, on recommendation of President T. C. Osborn, to take clinical notes of all cases of pneumonia coming under their observation, with especial reference to prodromata, duration and termination; the presence of herpetic eruption on the lips, etc., and, if present, what significance said eruption had as a prognostic indication; to report to the Society once a month the results of their observation, a record to be kept of such notes, and a tabulated report to be made at annual meeting.

The following officers were elected, each for one year: President, Dr. C. C. Francis; First Vice-President, Dr. G. B. Colby; Second Vice-President, Dr. J. S. Wagley; Secretary-Treasurer, (ex-President) T. C. Osborn; Censors, Drs. Osborn, Keating and Wagley.

The annual message of the President, Dr. T. C. Osborn, was delivered. On motion, a copy of the message was voted to DANIEL'S TEXAS MEDICAL JOURNAL for publication.*

The subject of catarrhal fever, as the term has been applied to the continued form of fever prevailing in Texas, was then discussed. Dr. Francis led the discussion, and after giving several reasons for his belief, thought the term a misnomer.

Dr. J. L. Wagley concurred with Dr. Francis in opinion that the term was not applicable to our typho-malarial fever, but was mildly deserving consideration in many of the cases met in this section, of a continued form of fever which never developed typhoid conditions, only so far as the hyperemic condition of the mucous membrane was concerned, which, on account of frequent discharges of mucus from the bowels, gave a slight significance of its catarrhal nature.

Dr. T. C. Osborn concurred with the others that it was a misnomer, and unworthy of discussion, only as an emphatic expression on the part of the Society denying the assertions made by a writer in the medical journals, endeavoring to change the old name, typho-malarial, to catarrhal fever. He did not deny that there was a catarrhal fever, and cited influenza as a typical form of it, and perhaps the asthmatic condition known as hay fever might also be clearly classed as catarrhal. The typho-malarial, or malarial typhoid fever, as he was accustomed to recognize it, was as appropriately named as any other disease, and required

*On account of an unusual press upon the columns of the JOURNAL for the past few months, the address has not yet been published. It will appear at an early date, and the attention of our readers will be called to it. It is of unusual interest and practical importance.—ED.

no change in its title to make it better understood and more amenable to treatment. If the downward flow, as the term catarrhal strictly meant, was to be used outside the fever of influenza, he could see no reason why dysentery and diarrhœa were not as much interested in the new application as any other disease on the necrological shelves.

Dr. Menefee concurred with the others in denying that our continued form of fever was in any sense catarrhal.

Dr. J. L. Wagley reported a case of inflammatory croup, which he had recently encountered in his practice, the patient dying on the —— day of the disease, in spite of the best directed treatment. Statistics, he said, proved that 90 per cent. of such cases prove fatal in from one to five days. He had laid himself out to adopt the best treatment in this case that was advised by the most eminent authorities, only to see the same result as had been everywhere observed.

The essayist being absent, his name was continued to the next meeting.

A motion was adopted to make croup the snbject for the next discussion, Dr. J. L. Wagley leading.

The meeting then adjourned to meet again at 2 o'clock p. m., on Thursday, December 10th.

T. C. OSBORN, M. D., Sec'y J. C. M. S.

